# Functional roles of Keratin 6A in disease pathogenesis across cancer and skin disorders

**DOI:** 10.3389/ebm.2026.10845

**Published:** 2026-01-21

**Authors:** Yanyan Su, Shudong Su, Min Li, Zhixia Zhang, Shiyi Zhang, Caixia Fan, Wei Luo, Shuming Guo

**Affiliations:** 1 Nursing College of Shanxi Medical University, Taiyuan, China; 2 Department of Nursing, Linfen Central Hospital, Linfen, China; 3 Department of Orthopaedics, People’s Hospital of Linfen City, Linfen, China; 4 Department of Nursing, Changzhi Medical College, Changzhi, China; 5 Department of Gastroenterology, Linfen Central Hospital, Linfen, China; 6 Department of Thyroid and Neck Tumor, Tianjin Medical University Cancer Institute and Hospital, National Clinical Research Center for Cancer, Key Laboratory of Cancer Prevention and Therapy, Tianjin’s Clinical Research Center for Cancer, Tianjin, China

**Keywords:** cancer, dermatoses, drug resistance, epithelial–mesenchymal transition, KRT6A, pachyonychia congenita, therapeutic biomarker

## Abstract

Keratin 6A (KRT6A) is an epithelial-specific type II keratin localized within cytoskeletal intermediate filaments and functions in cooperation with KRT16/17 to maintain epidermal homeostasis and tissue repair. Accumulating evidence highlights its multifaceted roles in cancer. Aberrant KRT6A expression promotes cell cycle progression, epithelial–mesenchymal transition, migration, and invasion, thereby driving tumor initiation and metastasis, although tumor-suppressive effects have been observed in specific contexts. Mechanistically, KRT6A regulates adhesion, cytoskeletal remodeling, and critical signaling pathways, thereby reshaping tumor immunity and metabolism to facilitate immune evasion and metabolic dysregulation. Elevated KRT6A expression is strongly associated with resistance to chemotherapy, targeted therapy, and radiotherapy. Therapeutic approaches targeting KRT6A include nucleic acid-based interventions, protein degradation strategies, inhibition of upstream regulatory pathways, and combinatorial regimens to overcome drug resistance. Clinically, KRT6A has emerged as both a diagnostic and prognostic biomarker, supporting treatment monitoring and enhancing predictive models for risk stratification and individualized outcome evaluation. Beyond oncology, mutations in KRT6A underlie pachyonychia congenita, and its dysregulation contributes to epidermal hyperproliferative disorders such as psoriasis. Overall, systematic elucidation of the structure–function–pathway–clinical axis of KRT6A offers new opportunities for precision medicine and supports its potential as a therapeutic target in cancer management.

## Impact statement

Keratin 6A (KRT6A) has emerged as a stress-inducible keratin with multifaceted roles that extend beyond structural support. Its dysregulation is increasingly recognized as a driver of cancer progression, treatment resistance, and poor prognosis, while pathogenic mutations cause inherited skin disorders such as pachyonychia congenita. Although several studies and reviews have broadly examined keratin biology and the contextual roles of K6 proteins in cancer, comprehensive synthesis specifically integrating emerging mechanistic and clinical evidence on KRT6A across both cancer and dermatologic diseases remains limited. This review integrates recent advances in the molecular, cellular, and clinical aspects of KRT6A, highlighting its contributions to cell proliferation, plasticity, immune regulation, and cytoskeletal dynamics. By bridging findings from oncology and dermatology, we provide insights into KRT6A as both a pathogenic mediator and a potential therapeutic biomarker. This work reframes KRT6A from a structural keratin to a disease-relevant effector, thereby informing future mechanistic research and translational applications.

## Introduction

Keratin 6A (KRT6A) is a member of the type II intermediate filament (IF) protein family, which plays a vital role in maintaining cytoskeletal integrity and epithelial tissue homeostasis. KRT6A, together with its isoforms KRT6B and KRT6C, forms part of the keratin 6 subfamily, which is primarily expressed in stratified epithelia and is essential for processes such as squamous epithelial differentiation and epidermalization [1–3].

Structurally, keratins assemble into obligate heteropolymers composed of one type I (acidic, low molecular weight) and one type II (basic to neutral, high molecular weight) keratin [1, 4, 5]. KRT6A shares the tripartite architecture typical of IF proteins, consisting of a non-helical head domain (residues 1–162), a central α-helical rod domain (163–476), and a non-helical tail domain (477–564). The rod domain is subdivided into several coiled-coil segments interspersed with linker regions, while the head domain contains an intrinsically disordered region at its N-terminus (residues 1–23) [6, 7].

Genetic mutations in KRT6A are implicated in various inherited skin disorders, such as pachyonychia congenita type I (PC-1) and focal non-epidermolytic palmoplantar keratoderma, highlighting its functional importance in epithelial integrity [8]. More recently, dysregulation of KRT6A has been linked to carcinogenesis, potentially via effects on mechanotransduction, cytoskeletal dynamics, and stress responses. KRT6A overexpression has also been associated with tumor aggressiveness and poor prognosis across several cancers [9]. Although prior studies and reviews have discussed keratin family biology and the roles of K6 proteins, including K6A, in cancer, analyses that integrate emerging mechanistic and clinical evidence on KRT6A across both cancer and dermatologic diseases remain limited [10–12]. Notably, many of these mechanistic insights are based on limited or single-study observations and therefore require further validation before firm conclusions can be drawn.

In this review, we comprehensively summarize current findings regarding the dual roles of KRT6A in both malignant and non-malignant epithelial diseases. Specifically, we focus on its contributions to tumor initiation, progression, metastasis, metabolic reprogramming, drug resistance, immune modulation, and prognostic relevance. Furthermore, we discuss the molecular mechanisms underlying KRT6A mutations in dermatoses and explore the potential of KRT6A-targeted strategies as future therapeutic options.

The literature included in this review was identified primarily through comprehensive searches of PubMed, supplemented by key studies published by major academic publishers (e.g., Elsevier, Wiley, and Springer Nature). Several relevant mechanistic studies were additionally retrieved through manual searches of CNKI. The search covered publications up to May 2025, with two newly published studies (June and October 2025) incorporated during revision to maintain currency. As this is a narrative review, no formal inclusion or exclusion criteria were applied; however, priority was given to influential and methodologically robust studies. In addition, we assessed key methodological features of the included studies, including sample size and whether *in vitro* findings were validated in animal models or human specimens, to better contextualize the strength of the available evidence.

## Roles of KRT6A in cancer

Several members of the keratin family, including KRT8, KRT17, KRT18, and KRT6A, play critical roles in cancer biology. Dysregulation of their expression—whether through overexpression or downregulation—is strongly linked to cancer initiation, progression, and metastasis. However, the strength of evidence varies considerably across tumor types and study designs, much of the mechanistic evidence remains preliminary, with several observations derived from single-cell line or *in vitro* studies lacking *in vivo* validation. Although mutations in keratin genes are relatively rare, they have been implicated in the development and advancement of certain cancers.

## Pro-tumorigenic role of KRT6A

Aberrant expression of KRT6A has been reported across multiple cancer types, often correlating with poor clinical outcomes. Overexpression of KRT6A has been documented in various malignancies, including cutaneous melanoma (CM) [13, 14], head and neck squamous cell carcinoma (HNSCC) [15], non-small cell lung cancer (NSCLC) [16–18], lung adenocarcinoma (LUAD) [19–23], bladder cancer [24], pancreatic adenocarcinoma (PAAD) [25], pancreatic ductal adenocarcinoma (PDAC) [26, 27], triple-negative breast cancer (TNBC) [28], colon adenocarcinoma (COAD) [29], and oral squamous cell carcinoma [30].

For example, Enzyme-linked immunosorbent assay (ELISA) analysis of 54 matched tumor and margin samples from HNSCC patients revealed significantly elevated KRT6A protein levels in tumor tissues compared to surgical margins, with smokers exhibiting higher tumor KRT6A expression than non-smokers [15]. Similarly, Reverse transcription–quantitative polymerase chain reaction (RT–qPCR) analysis of 75 NSCLC specimens showed that KRT6A was significantly upregulated in tumors relative to adjacent normal tissues. This upregulation correlated with advanced Tumor–Node–Metastasis (TNM) stage, lymph node and distant metastasis, and daily smoking status. Moreover, high KRT6A expression predicted poorer prognosis in NSCLC smokers, as demonstrated by survival and Cox proportional hazards (Cox) regression analyses [17]. Although these findings suggest a potential link between KRT6A expression and tumor aggressiveness, the evidence is largely correlational, and the extent to which KRT6A contributes causally to disease progression remains uncertain.

Functional studies provide additional but still preliminary support for a pro-tumorigenic role. *In vivo* experiments using nude mice injected with A549 lung cancer cells showed that short hairpin RNA (shRNA)-mediated knockdown of KRT6A significantly reduced tumor volume and weight compared to controls. Immunohistochemical staining indicated decreased Ki67-positive proliferative cells in KRT6A-knockdown tumors, consistent with *in vitro* findings demonstrating impaired tumor cell proliferation [17]. While these findings indicate that KRT6A may influence proliferation in specific experimental contexts, they are derived from single-cell-line models with relatively small sample sizes and require validation across additional systems and in human tissues.

Genetically, a novel tumor-specific variant of KRT6A (c.1048_1049delGGinsCG, p.Ala350Arg) was reported in a single hepatocellular carcinoma (HCC) patient. Although this mutation was absent from major genomic databases such as Single Nucleotide Polymorphism database (dbSNP) and Catalogue Of Somatic Mutations In Cancer (COSMIC), indicating it may represent a rare or previously uncharacterized alteration, its functional and clinical significance remains speculative given the single-case evidence and lack of experimental validation [31]. In addition, KRT6A mutations (e.g., c.745T>C) have been recurrently detected in peripheral blood granulocytes of Philadelphia-negative myeloproliferative neoplasm (MPN) patients with secondary cancers (SCs). Unlike the predominantly solid tumor-associated KRT6A mutations cataloged in COSMIC, this observation raises the possibility of KRT6A involvement in the hematopoietic compartment. OncodriveCLUST, a clustering-based driver mutation analysis algorithm, further indicated a stronger mutation-clustering signal for KRT6A than for the canonical JAK2V617F mutation in MPN-SC granulocytes, suggesting a potential inflammation-associated mechanism in SC pathogenesis [32]. However, because clustering-based driver prediction relies on computational modeling and is sensitive to sample size and algorithmic assumptions, these mutation-related findings should be considered preliminary. Although they suggest potential oncogenic or inflammation-associated roles for KRT6A mutations, the evidence remains limited and largely associative, underscoring the need for validation in larger cohorts and through mechanistic studies.

Overall, the current understanding of KRT6A’s pro-tumorigenic potential is shaped predominantly by *in vitro* studies, limited animal experiments, and bioinformatic analyses. While available data indicate possible roles in promoting malignant phenotypes, stronger evidence from mechanistic studies and large, well-characterized clinical cohorts is needed to clarify whether KRT6A acts as a functional driver, a context-dependent modulator, or simply a biomarker of tumor progression.

## Role of KRT6A in cancer cell metastasis

Building upon its established role in tumor growth, KRT6A has also been implicated in cancer cell migration, invasion, and metastasis across various cancer types. *In vitro* studies have demonstrated that KRT6A knockdown significantly reduces migratory and invasive capabilities in several cancer cell lines. For instance, small interfering RNA (siRNA)-mediated silencing of KRT6A in A549 lung cancer cells inhibited both migration (wound-healing assay) and invasion (Transwell assay) [18]. Similar findings were observed in HCC827 lung cancer cells. KRT6A knockdown reduced cell viability and proliferation, as demonstrated by the Cell Counting Kit-8 (CCK-8), colony formation, and 5-ethynyl-2′-deoxyuridine (EdU) assays. It also suppressed cell invasion [20]. In colon cancer HCT116 cells, knockdown of KRT6A impaired proliferation, migration, and invasion, while its overexpression enhanced these malignant phenotypes. DLD-1 cells with KRT6A overexpression exhibited stronger migration and three-dimensional (3D) invasive activity, particularly at the tumor invasive front, correlating with tumor budding and poor differentiation [33, 34]. These phenotypic effects, however, have been demonstrated primarily in cell-line models, and their relevance in more physiological systems remains to be determined.

KRT6A also modulates key signaling pathways involved in metastasis. In NSCLC cell lines, KRT6A may act downstream of lysine-specific demethylase 1 (LSD1) and promote invasion through c-MYC (MYC proto-oncogene protein) and MYCN (MYC proto-oncogene, neuroblastoma-derived)-driven upregulation of glucose-6-phosphate dehydrogenase (G6PD), thereby potentially activating the pentose phosphate pathway (PPP) [16]. Additionally, it has been reported to promote radioresistance, invasion, and metastasis in lung cancer, with pathway enrichment analyses suggesting possible involvement of the p53 signaling pathway [18]. In nasopharyngeal carcinoma cell models, KRT6A silencing downregulated matrix metalloproteinases-2/9 (MMP-2/9) and β-catenin signaling components, while increasing E-cadherin and tissue inhibitor of metalloproteinases-2 (TIMP-2). These molecular changes are consistent with a reversal of epithelial–mesenchymal transition (EMT). Activation of the Wnt/β-catenin pathway rescued these effects, suggesting that KRT6A may promote invasion and metastasis through β-catenin signaling [35]. Nevertheless, most of these pathway connections are inferred from single *in vitro* studies and enrichment analyses, and causal relationships remain to be experimentally validated.

KRT6A may be involved in EMT and cancer stem cell (CSC) maintenance. In lung adenocarcinoma (LUAD) cells, KRT6A knockdown increased epithelial markers (E-cadherin, β-catenin) and reduced mesenchymal markers (N-cadherin, vimentin). It also significantly decreased the CSC subpopulation (CXCR4^high/CD133^high) and colony-forming capacity, suggesting a potential role in EMT and CSC-associated phenotypes [19]. While these findings are mechanistically suggestive, they are derived almost entirely from LUAD cell models, and their generalizability to other cancer types remains unclear.

Beyond lung and colon cancer, KRT6A may contribute to bladder cancer progression, partly as a target of microRNA-31-5p (miR-31-5p). Low levels of this microRNA result in upregulation of KRT6A, promoting tumor cell proliferation, adhesion, and invasion [24]. In gastric adenocarcinoma, mitogen-activated protein kinase 1 (MAPK1) was shown to transcriptionally regulate KRT6A expression, facilitating AGS (human gastric adenocarcinoma) cell motility and invasion [36]. Similarly, dexamethasone-induced upregulation of KRT6A enhanced pancreatic cancer cell migration and invasion [37]. Importantly, these regulatory interactions arise from isolated studies across different cancer types, and their generalizability remains unclear.

Transcriptomic and proteomic analyses suggest that KRT6A exhibits divergent associations with melanoma progression. Elevated KRT6A expression in primary melanomas was associated with thinner tumors (Breslow thickness) but poorer survival in metastatic disease. As part of the epidermal differentiation complex (EDC), KRT6A expression correlated—based on transcriptomic and reverse-phase protein array (RPPA) analyses—with EMT signatures and activation of mitogen-activated protein kinase kinase (MEK), epidermal growth factor receptor (EGFR), and activating transcription factor-2 (ATF2) pathways. These findings suggest that KRT6A may contribute to molecular networks that facilitate metastasis and invasion [14]. Moreover, these associations are primarily correlative—based on transcriptomic and RPPA analyses—and functional evidence supporting direct mechanistic involvement remains limited.

**FIGURE 1:**
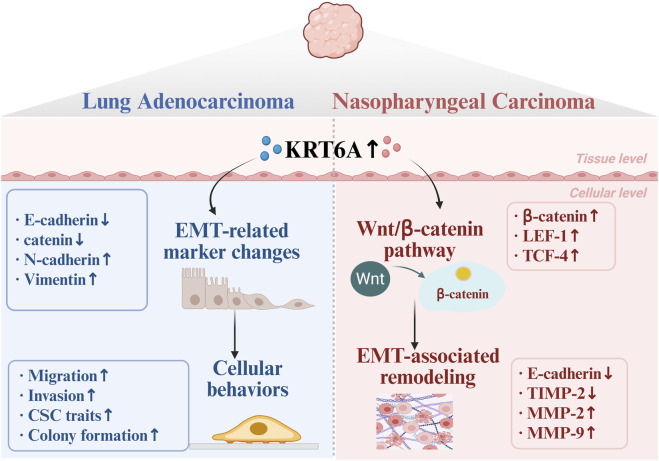
Schematic illustration of the role of KRT6A in regulating epithelial–mesenchymal transition (EMT) and malignant progression in lung adenocarcinoma and nasopharyngeal carcinoma. Accumulating evidence indicates that KRT6A regulates EMT-associated molecular alterations and malignant progression of cancer cells in a context-dependent manner. In lung adenocarcinoma, alterations in KRT6A expression are closely associated with dynamic changes in epithelial and mesenchymal markers, accompanied by phenotypic plasticity and enhanced invasiveness of cancer cells. In nasopharyngeal carcinoma, KRT6A is proposed to promote EMT-related phenotypic remodeling through activation of the Wnt/β-catenin signaling pathway. This schematic summarizes the currently available experimental and clinical evidence and highlights putative molecular mechanisms that require further experimental validation.

Collectively, these findings suggest that KRT6A may function as a multifunctional regulator of tumor metastasis, operating through EMT, CSC regulation, and diverse oncogenic pathways, as illustrated in Figure 1, although most supporting evidence comes from *in vitro* studies, single-model systems, or correlative transcriptomic analyses, highlighting the need for further mechanistic and *in vivo* studies to validate these proposed roles.

## Potential anti-tumor roles of KRT6A

While numerous studies support the pro-tumorigenic role of KRT6A, a few isolated reports suggests it may exert anti-tumor effects under certain contexts. In LUAD, KRT6A overexpression inhibited cell proliferation, migration, and invasion, as shown by CCK-8, colony formation, wound-healing, and Transwell assays. Furthermore, immunohistochemical analysis revealed that higher KRT6A protein levels were associated with improved patient prognosis [38]. These findings, while informative, are based primarily on *in vitro* assays and a commercially sourced tissue microarray, and have not yet been independently validated or confirmed *in vivo*, making their broader relevance uncertain.

Interestingly, a prior study from the same research group reported that elevated KRT6A mRNA expression predicted poor prognosis in LUAD patients based on analyses of public transcriptomic datasets [39]. This discrepancy between mRNA and protein-level findings suggests possible post-transcriptional regulatory mechanisms—most notably, microRNA-mediated suppression of translation. These findings highlight the need for further investigation into the context-dependent and regulatory complexity of KRT6A function in cancer.

In addition to post-transcriptional regulation, several biological factors may contribute to the context-dependent behavior of KRT6A. First, isoform-specific functions may drive divergent phenotypes, as distinct KRT6A splice variants could engage different cytoskeletal partners or signaling pathways. Second, tumor microenvironmental influences—including stromal composition, immune infiltration, and extracellular stress cues—may alter KRT6A activity or subcellular localization, thereby shaping its functional output. Third, variation in protein stability or post-translational modifications such as phosphorylation or ubiquitination may further fine-tune KRT6A signaling across tissues or disease stages. Overall, discrepancies between transcript-level and protein-level observations likely reflect multilayered regulatory complexity, underscoring the need to study KRT6A within defined cellular and microenvironmental contexts.

## Role of KRT6A in cancer immunity

Although the immune-related functions of KRT6A remain largely unexplored, several correlative studies suggest a potential link between KRT6A expression and the tumor immune microenvironment, particularly tumor-associated macrophages (TAMs). In pancreatic ductal adenocarcinoma (PDAC), KRT6A expression shows a strong positive correlation with the TAM marker integrin alpha M (ITGAM/CD11b) (Pearson correlation coefficient, r = 0.95). Co-localization of KRT6A and ITGAM in tumor tissues was reported based on immunofluorescence and immunohistochemistry (IHC); however, these findings are descriptive in nature and do not establish cell-type specificity, functional interaction, or causal relationships. Gene co-expression network analysis further suggested that KRT6A may be linked to TAM-associated gene modules such as COL5A2, COL1A2, and SPARC, which are enriched in extracellular matrix remodeling, MAPK/Wnt signaling, and antigen presentation pathways [26]. Additionally, Cell type Identification By Estimating Relative Subsets Of RNA Transcripts (CIBERSORT)-based immune profiling indicated that high KRT6A expression in PDAC tissues is associated with increased infiltration of M2-polarized macrophages, supporting a potential link with an immunosuppressive microenvironment [40]. Taken together, these findings point to a potential association between KRT6A and TAM-related immunosuppression in PDAC, although mechanistic causality has not yet been established.

Beyond macrophages, KRT6A may also influence innate immune responses. It has been implicated in the regulation of antimicrobial peptides (AMPs) [41], which could indirectly affect immune cell recruitment. Moreover, in granulocytes, ginsenoside Rg1 treatment was shown to upregulate KRT6A along with laminin subunit gamma-2 (LAMC2), desmocollin-2 (DSC2), and FosB proto-oncogene (FOSB), reversing noradrenaline-induced immunosuppression and restoring their cancer-killing activity [42]. These observations were made in specific experimental systems, and their relevance to PDAC biology or *in vivo* immunity remains to be clarified.

Together, these studies suggest that KRT6A may influence tumor progression not only through intrinsic cancer cell pathways but also through interactions with the tumor immune microenvironment. Nevertheless, current evidence is preliminary, largely correlative, and highly context-specific. Definitive mechanistic studies—particularly those examining macrophage–tumor cell crosstalk, cell-type specificity, and *in vivo* immune modulation—are needed to determine whether KRT6A functions as an immune regulator or represents a potential target in PDAC immunotherapy.

## Role of KRT6A in tumor metabolism

Although most studies focus on the structural and signaling functions of KRT6A, emerging evidence suggests its possible involvement in tumor metabolic reprogramming, although current findings remain limited and largely model-specific. In lung adenocarcinoma A549 cells, KRT6A expression increases under cobalt chloride (CoCl_2_)-induced hypoxia in parallel with elevated hypoxia-inducible factor 1-alpha (HIF1A) levels, whereas this pattern is not observed in PC9 cells, indicating a potential cell-line–specific response. HIF1A is a master regulator of cellular adaptation to hypoxia and a key driver of metabolic shifts in tumors, and this context raises the possibility that KRT6A expression may be linked to hypoxia-related cellular responses [23]. Nevertheless, the evidence is derived from a single *in vitro* system, and no studies have investigated whether KRT6A directly modulates HIF1A activity or downstream metabolic pathways.

In addition, a single study suggests that KRT6A may be involved in metabolic reprogramming through regulation of the pentose phosphate pathway (PPP). Experiments in non–small cell lung cancer (NSCLC) cell lines suggest that KRT6A, acting downstream of LSD1, may enhance c-MYC/MYCN-mediated transcription of glucose-6-phosphate dehydrogenase (G6PD), the rate-limiting enzyme of the PPP. Such changes could potentially increase nicotinamide adenine dinucleotide phosphate (NADPH) production and anabolic biosynthesis, thereby supporting tumor growth, invasion, and redox homeostasis [16]. Nonetheless, these findings are primarily based on *in vitro* NSCLC cell-line experiments and currently lack *in vivo* validation. Moreover, the proposed LSD1–KRT6A–MYC–G6PD axis is supported by only one study and has not yet been corroborated by metabolic flux assays or clinical evidence, leaving its broader relevance uncertain.

Taken together, these findings suggest that KRT6A may influence cancer cell metabolic pathways, potentially involving hypoxia-related signaling and PPP activity. However, these connections are primarily supported by *in vitro* evidence, and further research is needed to determine whether KRT6A functions as a broader metabolic modulator across different tumor types.

## Role of KRT6A in drug resistance

Drug resistance remains a major challenge in effective cancer therapy, and emerging evidence suggests that KRT6A expression is associated with reduced responsiveness to both chemotherapeutic and targeted agents in several cancer contexts. However, the mechanistic basis of this association remains incompletely understood. In NSCLC, *in vitro* experiments showed that KRT6A knockdown in H1299 and HCC827 cells increased their sensitivity to mitoxantrone and oxaliplatin, indicating that KRT6A may modulate chemotherapy responsiveness [43]. Similarly, in cisplatin-resistant cervical cancer cells (SiHa/DDP), downregulation of KRT6A impairs cell proliferation, promotes apoptosis, and enhances cisplatin sensitivity, implicating KRT6A as a potential therapeutic target to reverse platinum resistance [44]. However, these findings rely on short-term *in vitro* assays, and whether KRT6A directly modulates chemoresistance mechanisms remains unclear.

Beyond traditional chemotherapy, KRT6A has also been implicated in resistance to targeted therapies. In liver kinase B1 (LKB1)-deficient, KRAS-mutant NSCLC, studies in mouse models and organoids show that epigenetic activation of the C40 enhancer induces ΔNp63 and downstream KRT6A expression, driving adeno-to-squamous transition (AST). This phenotypic switch enables tumors to bypass KRAS dependency and confers resistance to KRAS inhibitors such as Adagrasib (G12C) and MRTX1133 (G12D). Consistently, patient biopsy data indicate that high KRT6A expression is associated with poor response and unfavorable prognosis [45]. Although the evidence is stronger due to cross-model consistency, direct functional validation of KRT6A as a driver of AST-mediated resistance is still limited.

Furthermore, KRT6A is identified as a core gene in a machine learning model for acquired resistance to EGFR-tyrosine kinase inhibitors (EGFR-TKIs; e.g., gefitinib, erlotinib, afatinib) and cetuximab, suggesting a potential broader involvement in resistance to anti-EGFR therapies [46]. As this conclusion is mainly based on computational modeling, experimental confirmation of KRT6A’s functional contribution to anti-EGFR resistance is still lacking.

Collectively, current evidence suggests that KRT6A may contribute to diverse forms of therapy resistance across multiple cancer types; however, most available data are derived from isolated *in vitro* studies or computational predictions. Robust *in vivo* experiments and mechanistic investigations are still lacking, and no causal link between KRT6A and drug resistance has been firmly established. Therefore, while KRT6A represents an intriguing candidate for therapeutic targeting, its functional role in treatment resistance requires substantially more experimental validation.

## Role of KRT6A in radiation resistance

KRT6A has been implicated in the development of radioresistance in non–small cell lung cancer (NSCLC), although current evidence remains limited and largely context-dependent. In A549-derived radiation-resistant cells (A549-RR), KRT6A expression is markedly elevated, and its silencing enhances radiosensitivity. Mechanistically, Western blot analysis shows that β-catenin levels decline after KRT6A knockdown, whereas β-catenin overexpression rescues both EMT phenotypes and radiation resistance, supporting the involvement of the Wnt/β-catenin–EMT axis. However, these mechanistic data are derived from *in vitro* assays only and have not yet been validated *in vivo* [47].

In support of these findings, The Cancer Genome Atlas (TCGA) RNA sequencing (RNA-seq) data from lung squamous cell carcinoma (LUSC) patients revealed KRT6A as the most significantly upregulated gene in the low radiosensitivity index (RSI) group compared to the high RSI group [48], although this correlation does not imply a causal relationship between KRT6A expression and radiosensitivity.

Additionally, KRT6A may influence radiation response by modulating pathways such as p53 signaling and G2/M cell-cycle regulation—both of which are linked to DNA damage repair and tumor cell survival under irradiation stress. These associations are derived from public database analyses, and the underlying mechanisms require further validation in cellular and animal models [18].

Taken together, existing evidence suggests that KRT6A expression is associated with reduced radiosensitivity in NSCLC and may serve as a potential biomarker of radiation response. However, whether KRT6A functions as a direct mediator of radioresistance or represents a surrogate marker of resistant tumor states remains unresolved. Further *in vivo* and mechanistic studies are required before KRT6A can be considered a viable therapeutic target in the context of radiotherapy.

## Targeting KRT6A: drugs and therapeutic strategies

Several studies have explored pharmacological approaches that may modulate KRT6A expression or function, although direct targeting of KRT6A remains largely conceptual and has not yet been broadly validated. Sinapine thiocyanate (ST) exerts anti-tumor activity in colorectal cancer (CRC) partly by suppressing the KRT6A/S100A2 axis, with S100A2 (a calcium-binding protein of the S100 family) serving as a downstream mediator. In CRC cell lines, ST decreases KRT6A mRNA and protein expression and inhibits cell proliferation and migration. *In vivo*, ST similarly reduces tumor growth in xenograft models. Rescue experiments further show that overexpression of KRT6A counteracts ST-induced inhibition of malignant phenotypes, supporting KRT6A as a functional target of ST. Consistent with these findings, elevated KRT6A/S100A2 expression correlates with poorer prognosis in CRC patients [49]. However, these findings are restricted to CRC models, and it remains unclear whether ST exerts comparable KRT6A-dependent effects in other tumor types or whether KRT6A is the primary molecular target of ST.

Taxifolin, a natural flavonoid, was identified as a candidate therapeutic agent through Connectivity Map (CMAP) analysis. This prediction was derived from a weighted gene co-expression network analysis (WGCNA) module in which KRT6A was one of 11 hub genes associated with poor prognosis across multiple cancer types [25]. However, this prediction is based on transcriptomic similarity rather than functional validation, and a direct mechanistic link between taxifolin and KRT6A regulation has not been experimentally confirmed.

Additionally, KRT6A has been reported as a gene associated with progression from Barrett’s esophagus (BE) to esophageal adenocarcinoma (EAC). Bioinformatics analysis using the Drug–Gene Interaction Database (DGIdb) predicted TD101, an siRNA previously used for pachyonychia congenita, as a potential KRT6A-targeting therapeutic agent, although its efficacy in EAC remains to be experimentally validated [50].

Collectively, existing studies nominate KRT6A as a potential therapeutic vulnerability; however, no KRT6A-directed agents have yet demonstrated consistent efficacy across cancer models or advanced toward clinical translation. Rigorous mechanistic studies, validation in diverse tumor systems, and preclinical evaluations are required to determine whether KRT6A represents a viable and druggable target in oncology.

## KRT6A as a diagnostic and therapeutic biomarker in cancer

KRT6A is emerging as a versatile biomarker across multiple cancer types. Its expression is significantly higher in lung squamous cell carcinoma (LSCC) compared to lung adenocarcinoma (LUAD), making it a potential diagnostic marker to distinguish these lung cancer subtypes [39, 51–53]. Proteomic analyses of exhaled breath condensate (EBC) further reported elevated KRT6A levels in lung cancer patients relative to controls, smokers, and Chronic Obstructive Pulmonary Disease (COPD) patients, suggesting promise for noninvasive early detection [54]. However, this conclusion is based on a small cohort from a single center, which constrains its broader generalizability. Moreover, KRT6A expression correlates with smoking exposure and has been proposed as a potential early diagnostic biomarker in smoking-related NSCLC [17], although prospective validation is still lacking.

Beyond lung cancer, KRT6A has also been implicated in other malignancies. It serves as a characteristic biomarker for basal-subtype bladder cancer [55, 56], and its expression is significantly higher in progesterone receptor B–high (PRB-H) breast tumors than in progesterone receptor A–high (PRA-H) tumors [57]. These associations, however, are mostly derived from retrospective datasets and have not been systematically examined in larger, clinically annotated cohorts.

Collectively, available evidence suggests that KRT6A may serve as a diagnostic or subtype-associated biomarker in several cancer contexts. Nevertheless, its clinical utility remains exploratory, and rigorous validation in large, multicenter studies—ideally with standardized assays and longitudinal follow-up—will be required before KRT6A can be adopted for routine clinical use.

## Prognostic biomarker correlation with poor outcomes

KRT6A expression has been consistently reported to correlate with tumor progression and poor prognosis across multiple cancer types, although the strength and consistency of these associations vary by tumor context and disease stage. In non-small cell lung cancer (NSCLC), particularly lung adenocarcinoma (LUAD), KRT6A overexpression is associated with aggressive clinicopathological features such as lymph node metastasis, advanced T stage, and worse overall survival (OS) and recurrence-free survival (RFS) [16, 18, 19, 23]. However, this correlation appears less clear in early-stage NSCLC [51]. In conjunction with LSD1, KRT6A may serve as a candidate prognostic indicator, with potential implications for therapeutic targeting in NSCLC [16]. To provide an integrated snapshot across tumor types, we compiled representative studies reporting KRT6A expression and its prognostic associations in major cancers (Table 1).

**TABLE 1:** KRT6A expression and prognostic significance across cancer types.

Cancer	Sample size	Expression tumor vs. normal	Prognostic of high KRT6A	OS	
				HR (95% CI)	p-value
NSCLC [13]	TCGA: 483 LUAD/486 LUSC Clinical IHC: 30	↑ mRNA & protein	Worse OS	1.491	0.016
LUAD [20]	TCGA: 535 tumors/59 normals	↑	Worse OS/DSS/PFI	1.156 (1.097–1.223)	<0.001
PDAC [34]	Clinical IHC: 130 TCGA-PAAD: 177	↑	Worse OS/DFS	2.704 (1.374–5.320)	0.004
PDAC [24]	TCGA-PAAD: 178 tumors/171 normals 7 paired cases	↑	Worser OS	1.09 (1.04–1.14)	0.00037
CRC [31]	Clinical IHC: 142 348 validation	↑Invasive front vs. center	Worse OS/DSS/PFS	3.081 (1.653–5.740)	0.0004
CM [10]	GEO: 99 CM vs 45 nevi TCGA: 475 CM Clinical IHC: 31 CM vs 31 nevi	↑Melanoma vs. nevus	Worse OS	1.07 (1.037–1.103)	<0.001
BLCA [21]	TCGA: 404 tumors vs. 28 normals	↑	Worse OS	1.42 (1.05–1.93)	0.023

Abbreviations: NSCLC, non-small cell lung cancer; LUAD, lung adenocarcinoma; PDAC, pancreatic ductal adenocarcinoma; CRC, colorectal cancer; CM, cutaneous melanoma; BLCA, bladder cancer; LUSC, lung squamous cell carcinoma; PAAD, pancreatic adenocarcinoma; TCGA, The Cancer Genome Atlas; GEO, Gene Expression Omnibus; IHC, immunohistochemistry; OS, overall survival; DSS, disease-specific survival; DFS, disease-free survival; PFS, progression-free survival; PEI, Progression-Event Interval; HR, hazard ratio.

“↑” Indicates higher KRT6A expression in tumor tissues or in aggressive tumor subsets.

References: NSCLC [13]; LUAD [20]; PDAC [24, 34]; CRC [31]; CM [10]; BLCA [21].

In pancreatic ductal adenocarcinoma (PDAC), elevated KRT6A expression is an independent predictor of poor OS and disease-specific survival (DSS), particularly in poorly differentiated tumors, reflecting its link to malignant phenotypes and tumor aggressiveness [26, 27, 37, 58, 59]. KRT6A has also been linked to alterations in the tumor immune microenvironment, particularly through associations with TAM-related pathways [26], however, these observations are primarily correlative.

In colorectal cancer (CRC), KRT6A has been identified as an independent prognostic factor for OS, DSS, and progression-free survival (PFS), correlating with lymph node metastasis and tumor stage [29, 34]. Interestingly, metastatic microsatellite instability–high (MSI–H) CRC tumors show reduced KRT6A expression compared to non-metastatic cases, suggesting subtype-specific roles [60].

High KRT6A levels have been reported to associate with advanced disease and poor prognosis in melanoma [13] and bladder cancer [24]. In papillary thyroid microcarcinoma (PTMC), elevated expression of CK5/6 (encoded by KRT5/KRT6A) predicts central lymph node metastasis, supporting its utility as a predictive biomarker [61]. Furthermore, an aggressive basal-like tumor cell subpopulation expressing KRT6A, KRT5, and KRT17 correlates with poor outcomes in intrahepatic cholangiocarcinoma (ICC) [62].

Collectively, these studies suggest that KRT6A expression is frequently associated with adverse prognosis across diverse malignancies. Nevertheless, existing prognostic evidence is derived from heterogeneous study designs, retrospective cohorts, and variable analytical methodologies. As such, KRT6A should currently be regarded as a candidate prognostic biomarker rather than a validated clinical indicator, and prospective, standardized studies will be required to define its independent prognostic value and potential clinical utility.

## KRT6A as a core component of predictive risk models

Multigene prognostic models have emerged as valuable tools in predicting cancer outcomes alongside traditional clinicopathological parameters. KRT6A, which harbors mutations across various cancers [21], is frequently included in these models. However, its inclusion should be interpreted as a statistical contributor rather than definitive evidence of causal biological centrality.

In triple-negative breast cancer (TNBC), KRT6A is consistently incorporated into multiple prognostic signatures—such as a 6-gene risk model, a necroptosis-related 7-gene model, and a senescence-associated 4-gene model—where it serves as a high-risk indicator linked to poor survival [28, 63]. Notably, the necroptosis-related model not only predicts survival but also explores potential therapeutic stratification based on predicted drug-response patterns, as reflected by estimated half-maximal inhibitory concentration (IC50) differences for cisplatin and lapatinib. A corresponding nomogram further demonstrates reasonable discriminative performance (AUC >0.84) [64]. Nevertheless, these findings are derived from retrospective analyses and predictive modeling rather than prospective therapeutic validation.

In pancreatic ductal adenocarcinoma (PDAC), 5-gene models that include KRT6A predict overall survival independently. These signatures link KRT6A to immune regulation, vascular invasion, and aggressive squamous subtypes, providing further evidence for its role as a malignancy-associated factor [65, 66], but they remain primarily correlative and model-dependent.

In lung adenocarcinoma (LUAD), KRT6A is embedded in a wide array of predictive models—such as those related to fatty acid metabolism [20], autophagy [67], tumor microenvironment (LATPS) [68], genomic instability (GSAGI) [69], DNA damage repair (DDR) [70], lymph node metastasis [71], RNA modification (RMScore) [72], TEAD4 (TEA domain transcription factor 4)-related pathways [73], pyroptosis [74], liquid–liquid phase separation (LLPS) [75], and ferroptosis [76]. Across these models, high KRT6A expression or inclusion in high-risk groups consistently correlates with worse outcomes (OS, DFS, or RFS). Additionally, KRT6A appears in independent 5- and 13-gene models [77, 78]. The breadth of its inclusion likely reflects its strong correlation with aggressive tumor states rather than pathway-specific causality.

In colorectal cancer (CRC), KRT6A functions as a key component of a validated 5-gene prognostic signature, showing independent predictive power across The Cancer Genome Atlas (TCGA), GSE39582, and GSE17538 cohorts for overall survival (OS), disease-free survival (DFS), and disease-specific survival (DSS). Enrichment analysis links the KRT6A-containing signature to extracellular matrix remodeling, particularly collagen-containing matrix pathways, which are commonly associated with tumor progression [33].

Collectively, these studies indicate that KRT6A is frequently incorporated into multigene prognostic models and consistently associated with adverse clinical outcomes. However, most existing models are derived from retrospective datasets and employ heterogeneous feature-selection and validation pipelines, raising concerns regarding overfitting and cross-study comparability. Accordingly, KRT6A should be viewed as a recurrent statistical risk-associated marker rather than a validated standalone predictor, and prospective validation will be essential to establish its clinical utility.

## Emerging roles of KRT6A in HPV-related tumors and immunotherapy

Recent studies indicate that KRT6A, previously viewed mainly as a cytoplasmic structural keratin, can also localize to the nucleus in human papillomavirus 16 (HPV16)-positive cervical cancer cells. Nuclear KRT6A interacts with TEA domain (TEAD) transcription factors and is recruited to the HPV long control region, supporting E6/E7 transcription and promoting tumor cell proliferation. Loss-of-function and rescue-of-function studies indicate that KRT6A contributes to the maintenance of HPV E6/E7 expression [79]. Although these observations were generated in HPV16-positive squamous carcinoma cell lines and require validation across other high-risk HPV genotypes and primary tumor settings, they highlight an emerging interface between keratin biology and viral transcriptional programs, suggesting that aberrant KRT6A activity may contribute to HPV-driven carcinogenesis.

Pan-cancer analyses link KRT6A to immune checkpoint pathways. In syngeneic immunotherapy models, KRT6A expression increased in responders to anti-cytotoxic T-lymphocyte–associated antigen 4 (CTLA-4) therapy, but decreased in non-responders receiving combined anti-CTLA-4 and anti-programmed cell death protein 1 (PD-1) treatment, whereas no significant changes were observed with other immune checkpoint blockade (ICB) regimens. These observations suggest that KRT6A expression dynamics may reflect treatment-specific immune responses rather than serving as a universal immunotherapy biomarker.

In parallel, drug-response profiling (CTRP/GDSC) further showed that high KRT6A expression was associated with greater sensitivity to several EGFR/HER (ErbB) family inhibitors [12]. However, these associations are derived from large-scale pharmacogenomic correlations and do not establish a direct mechanistic role for KRT6A in modulating immune checkpoint efficacy or targeted therapy response.

Collectively, these emerging findings expand the functional landscape of KRT6A to include potential roles in HPV-driven oncogenesis and therapy-associated immune contexts. Nevertheless, most supporting evidence remains correlative or model-specific, and further mechanistic and clinical validation will be required to determine whether KRT6A functions as an active regulator or a context-dependent biomarker in immunotherapy and virus-associated cancers.

## KRT6A in dermatoses

KRT6A is a type II intermediate filament protein encoded by the KRT6A gene located on chromosome 12q13.13 [3]. Under normal conditions, KRT6A is selectively expressed in specialized epithelial tissues such as the palmar/plantar epidermis, nail bed, hair follicle, and oral mucosa [80, 81]. Upon epithelial injury, mechanical stress, or inflammatory stimulation, KRT6A expression is rapidly and robustly induced [82]. This response is mediated by signaling cascades involving pro-inflammatory cytokines (e.g., IL-1, TNF-α), growth factors, and transcriptional regulators (e.g., NF-κB, AP-1) [83]. However, the relative contribution of these pathways may vary depending on tissue context and experimental conditions.

Functionally, KRT6A enhances epithelial resilience against mechanical trauma and facilitates wound healing by supporting keratinocyte proliferation and migration [11, 84]. These roles are primarily supported by experimental models of skin injury and keratinocyte culture systems, and the extent to which they generalize across different epithelial tissues remains to be fully elucidated.

In pathological conditions, such as inflammatory skin diseases (e.g., psoriasis, lichen planus) and inherited keratin disorders, this inducible expression becomes dysregulated [3]. Aberrant KRT6A upregulation contributes to hyperproliferative phenotypes and epidermal barrier dysfunction [10]. Moreover, mutations in KRT6A are causally linked to pachyonychia congenita type I, a rare genodermatosis characterized by painful palmoplantar keratoderma, nail dystrophy, and oral leukokeratosis [85]. These findings underscore KRT6A’s pivotal role not only in epithelial homeostasis but also in the pathogenesis of diverse dermatoses.

## KRT6A in pachyonychia congenita

Pachyonychia Congenita (PC) is a rare autosomal dominant genodermatosis caused by mutations in genes encoding stress keratins, with KRT6A mutations accounting for approximately 30% of all PC cases (PC-K6a subtype) [85, 86]. According to data from the International Pachyonychia Congenita Research Registry (IPCRR), over 50 pathogenic variants of KRT6A have been documented—most of them being missense mutations (e.g., p.Asn171Lys, p.Arg162Pro), as well as deletions, insertions, and splice site alterations. These mutations predominantly affect the helix initiation and termination motifs critical for keratin filament assembly.

Mutant KRT6A proteins impair cytoskeletal integrity by disrupting filament organization, leading to cellular fragility and mechanical stress sensitivity [87]. The accumulation of misfolded keratin proteins triggers endoplasmic reticulum (ER) stress, activating c-Jun N-terminal kinase (JNK) and p38 mitogen-activated protein kinase (p38 MAPK) signaling pathways and promoting cellular stress responses [88]. In addition, mutant KRT6A induces dysregulated apoptosis via enhanced caspase-3 activation in basal keratinocytes [89]. Compensatory upregulation of KRT6A and KRT16 aggravates the pathology by driving keratinocyte hyperproliferation and impaired differentiation, contributing to disease progression [90].

Clinically, PC is characterized by a triad of focal palmoplantar keratoderma, hypertrophic nail dystrophy, and debilitating plantar pain. Other manifestations may include follicular hyperkeratosis, oral leukokeratosis, natal teeth, sebaceous cysts, hidradenitis suppurativa, hoarseness, and itching [85, 86, 91, 92]. Among patients with PC-K6a mutations, painful plantar keratoderma is often reported as the most debilitating and treatment-resistant symptom [91].

Currently, no definitive cure exists for PC, and management remains symptomatic. Conservative approaches include reducing plantar trauma, mechanical debridement, and application of topical keratolytic agents [93, 94]. Some patients benefit from oral retinoids [95], while botulinum toxin injections into the plantar region have been shown to alleviate pain, callus formation, and blistering [96–98]. Oral statins have also demonstrated efficacy in reducing callus thickness and pain levels in select patients [99–102].

Emerging targeted therapies are under exploration. A summary of representative KRT6A variants in PC-K6a and their mechanistic and therapeutic implications is provided in Table 2. These include small-molecule inhibitors, mechanistic target of rapamycin (mTOR) pathway modulators [103, 104], and gene therapy strategies such as small interfering RNA (siRNA) targeting KRT6A [105–107]. Notably, EGFR inhibitors (e.g., erlotinib, lapatinib) have been reported in individual cases to alleviate plantar hyperkeratosis and pain [108, 109]. Additionally, sunitinib, a multi-target tyrosine kinase inhibitor, was found to reduce KRT6A and serine protease inhibitor B1 (SERPINB1) expression *in vitro* by inhibiting extracellular signal-regulated kinase 1/2 (ERK1/2) and p38 MAPK signaling [110], suggesting a potential future therapeutic avenue. These findings are preliminary and largely based on case reports or experimental models, underscoring the need for systematic clinical evaluation.

**TABLE 2:** KRT6A mutations in pachyonychia congenita (PC-K6a): functional and therapeutic implications.

Variant (protein)	Frequency in PC	Functional impact	Therapeutic notes
p.Asn172del	10.9%	Disrupts helix‐initiation motif; induces cytoskeletal fragility	Symptomatic care; limited response to retinoids; Case-dependent EGFR/mTOR inhibitor responses; Emerging siRNA approaches; no mutation-specific evidence
p.Val181_Gln186del	3%	Alters L12 linker; reduces filament flexibility; weakens cytoskeletal network	
p.Glu472Lys	2%	Disrupts helix‐termination motif; impairs filament maturation	
p.Asn171Lys	1.6%	Destabilizes helix‐initiation motif; impairs filament assembly	
p.Leu469Pro	1.2%	Disrupts helix‐termination motif; impairs filament stabilization and maturation; induces cytoskeletal fragility	

Representative KRT6A variants in PC-K6a. Variant nomenclature follows HGVS guidelines; mutation data are referenced from the International Pachyonychia Congenita Research Registry. EGFR, epidermal growth factor receptor; mTOR, mechanistic target of rapamycin; siRNA, small-interfering RNA.

## KRT6A in psoriatic dermatitis

KRT6A expression is significantly upregulated in psoriasis-like dermatitis, contributing to disease development through multiple mechanisms [111–113].

**FIGURE 2:**
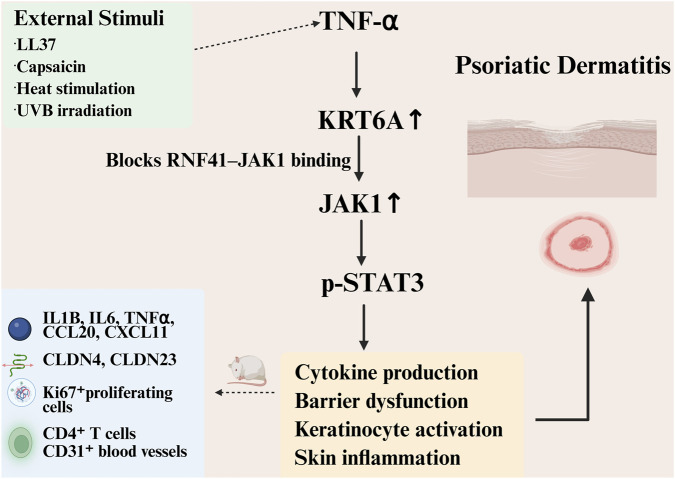
Schematic illustration of the KRT6A-mediated inflammatory signaling mechanism in psoriatic dermatitis. In psoriatic lesions and in the imiquimod (IMQ)-induced mouse model, KRT6A is markedly upregulated in epidermal keratinocytes in response to inflammatory stimuli. Elevated KRT6A may sustain activation of the JAK1–STAT3 signaling pathway by inhibiting RNF41-mediated ubiquitination and subsequent degradation of JAK1, thereby promoting the production of pro-inflammatory cytokines, aberrant activation of keratinocytes, impairment of skin barrier function, and amplification of cutaneous inflammation. This schematic model is primarily supported by evidence from *in vitro* and animal studies.

In clinical psoriatic specimens and imiquimod (IMQ)-induced mouse models, KRT6A is markedly overexpressed in epidermal keratinocytes. Functional experiments demonstrate that KRT6A knockdown attenuates inflammation, while KRT6A overexpression worsens pathological phenotypes. Mechanistically, *in vitro* evidence suggests that KRT6A may enhance inflammatory responses by activating the signal transducer and activator of transcription 3 (STAT3) signaling pathway, likely through the inhibition of ring finger protein 41 (RNF41)-mediated ubiquitination and degradation of Janus kinase 1 (JAK1), thereby sustaining STAT3 activation and promoting the expression of proinflammatory cytokines in keratinocytes [114], as depicted in Figure 2. Nonetheless, these mechanistic insights are based largely on *in vitro* assays, and the RNF41–JAK1 axis has not been validated *in vivo*.

In addition, narrow-band ultraviolet-B (NB-UVB) therapy, a standard treatment for psoriasis, significantly downregulates KRT6A gene expression in both the peripheral and central regions of psoriatic plaques. This downregulation is associated with the normalization of keratinocyte differentiation, suggesting that KRT6A may act as a mediator of abnormal keratinization in psoriasis. However, these transcriptomic findings are based on a limited sample size and remain correlative [115].

## Discussion

KRT6A, a stress-inducible type II keratin, plays a multifaceted and context-dependent role in both epithelial malignancies and dermatoses. In cancers, its overexpression is frequently associated with enhanced tumor aggressiveness, metastatic potential, poor prognosis, and resistance to standard therapies. Mechanistically, KRT6A contributes to malignant progression via promoting proliferation, migration, epithelial–mesenchymal transition (EMT), immune modulation, and cell death resistance. In contrast, pathogenic mutations in KRT6A underlie the inherited disorder pachyonychia congenita (PC), in which cytoskeletal disorganization, keratinocyte fragility, and ER stress result in severe skin phenotypes.

Current evidence indicates that KRT6A is subject to complex regulatory mechanisms at the transcriptional and post-translational levels, although our understanding of its upstream regulators, post-translational modifications (PTMs), and interactome remains incomplete. Proteomic insights suggest phosphorylation and possibly other PTMs may fine-tune KRT6A function in distinct pathophysiological contexts. However, comprehensive mapping of these modifications and their functional consequences is urgently needed.

From a translational perspective, KRT6A represents a promising candidate for both biomarker development and therapeutic targeting. In oncology, its incorporation into multi-gene prognostic models has improved patient stratification and therapeutic guidance, particularly in TNBC, LUAD, PDAC, and CRC. In hereditary skin diseases, topical or systemic delivery of RNA interference (RNAi) or small-molecule inhibitors targeting KRT6A or its downstream effectors offers potential therapeutic benefit.

Several limitations should be considered when interpreting the available evidence. First, many clinical investigations were conducted with relatively small patient cohorts and often lacked multi-center validation, limiting the generalizability of the findings. Second, mechanistic insights into KRT6A function largely rely on *in vitro* cell line models, with limited *in vivo* or clinical evidence to support biological relevance. Third, several mechanistic conclusions originate from single-study observations or correlative transcriptomic and bioinformatic analyses, which do not establish causality. Additionally, cancer-type heterogeneity and varied experimental conditions further complicate the interpretation and comparison of results.

Future research should focus on: (1) large-scale clinical validation of KRT6A as a diagnostic and prognostic biomarker; (2) elucidation of its molecular network and upstream regulatory pathways across disease types; (3) development of safe and efficient delivery systems for RNAi and antibody-based therapeutics; (4) integration of single-cell and spatial multi-omics technologies to delineate KRT6A-associated cellular programs with high resolution; (5) establishment of KRT6A knockout and conditional genetic models to dissect its context-dependent functions *in vivo*; and (6) initiation of early-phase clinical trials for KRT6A-targeted inhibitors to accelerate therapeutic translation. In addition, mechanistic studies on KRT6A’s role in immune regulation, ferroptosis, and tissue remodeling could uncover novel vulnerabilities for therapeutic exploitation.

**FIGURE 3:**
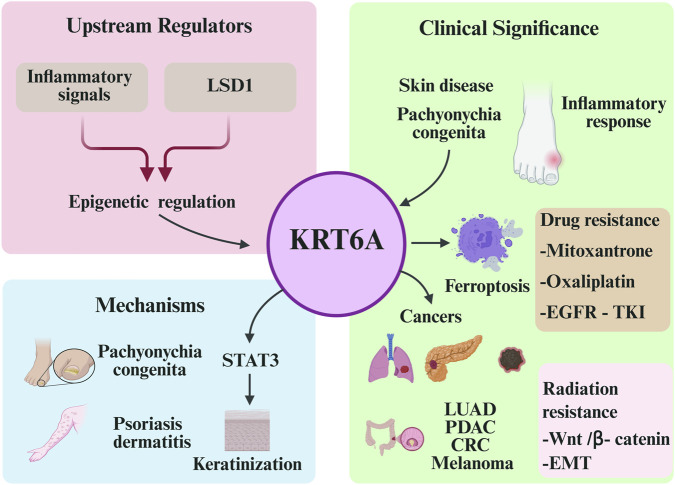
Schematic representation of the upstream regulation, molecular mechanisms, and clinical significance of KRT6A. Inflammatory signals (e.g., TNF-α, IL-1β) and the epigenetic regulator LSD1 can upregulate KRT6A expression. KRT6A is involved in multiple biological processes, including pachyonychia congenita and psoriatic dermatitis, partly via the STAT3 pathway, and is associated with ferroptosis and inflammatory responses. Clinically, KRT6A is closely linked to the development and progression of various cancers, and contributes to both chemotherapy and radiotherapy resistance. It holds potential as a diagnostic and prognostic biomarker.

A schematic summary of KRT6A’s upstream regulation, molecular mechanisms, and clinical significance is illustrated in Figure 3, providing an integrated visual overview that complements these conclusions. In conclusion, KRT6A functions as a central epithelial regulator with significant implications for disease pathogenesis and therapeutic intervention. Ongoing efforts to decode its regulatory mechanisms and disease-specific functions will pave the way toward KRT6A-based precision medicine strategies in both oncology and dermatology.
